# Early Recurrence of Pleomorphic-Type Anaplastic Pancreatic Carcinoma After Distal Pancreatectomy Causing Delayed-Onset Pancreatic Fistula: A Case Report

**DOI:** 10.7759/cureus.84316

**Published:** 2025-05-18

**Authors:** Masahiro Kobayashi, Masaru Matsumura, Yutaka Takazawa, Junichi Shindoh, Masaji Hashimoto

**Affiliations:** 1 Gastroenterological Surgery, Toranomon Hospital, Tokyo, JPN; 2 Pathology, Toranomon Hospital, Tokyo, JPN

**Keywords:** anaplastic cancer of pancreas, eus-fna, hepatic-bilio-pancreatic surgery, pancreatic cancer resection, pleomorphic cell, postoperative pancreatic fistula

## Abstract

Pleomorphic-type anaplastic carcinoma of the pancreas is a rare and highly aggressive histological subtype of pancreatic ductal carcinoma. It is characterized by rapid progression and a poor prognosis. Preoperative diagnosis is often challenging due to nonspecific imaging findings and the frequent absence of elevated tumor markers. We present a resected case of pleomorphic-type anaplastic carcinoma of the pancreatic tail, which showed early recurrence in the remnant pancreas, potentially associated with a delayed-onset pancreatic fistula.

A 63-year-old man presented with upper abdominal pain. Imaging revealed a cystic lesion in the pancreatic tail. Follow-up imaging showed enlargement of the lesion, and a retention cyst with possible underlying pancreatic carcinoma was suspected. Endoscopic ultrasound-guided fine-needle aspiration (EUS-FNA) was not performed due to concerns for cyst rupture. The patient underwent distal pancreatectomy with splenectomy. Histopathological examination confirmed pleomorphic-type anaplastic carcinoma. Although the drain was removed on postoperative day (POD) five due to low amylase levels in the drainage tube, a pancreatic fistula developed on POD 14, resulting in an intractable pancreatic fistula requiring persistent drainage. On POD 53, imaging revealed tumor recurrence in the remnant pancreas, along with peritoneal dissemination and right femoral bone metastasis. Retrospective evaluation of CT on POD 14 showed tumor recurrence compressing the main pancreatic duct, which was suspected to be the cause of the fistula. The patient declined further oncological treatment and died on POD 103.

This case highlights the diagnostic and therapeutic challenges of pleomorphic-type anaplastic carcinoma of the pancreas. Early postoperative recurrence can lead to pancreatic stump disruption and the development of intractable pancreatic fistula.

## Introduction

Anaplastic carcinoma of the pancreas is a rare and aggressive variant of pancreatic cancer, accounting for approximately 0.1% to 7% of all pancreatic malignancies globally, with variability depending on the population studied [1-3]. According to the Pancreatic Cancer Registry in Japan, the incidence is only 0.14% (38 of 27,335 cases), highlighting its rarity in this population [1]. It exhibits distinct clinical and pathological characteristics compared to conventional pancreatic ductal adenocarcinoma, and its prognosis is dismal [4].

Due to its aggressive clinical course and diagnostic difficulty, the high rates of local recurrence and distant metastasis following surgery present significant management difficulties. These features make this disease particularly noteworthy and warrant detailed reporting to raise clinical awareness.

We present a case of pleomorphic-type anaplastic carcinoma with distal pancreatectomy, followed by a delayed onset of postoperative pancreatic fistula likely caused by early recurrence in the remnant pancreas.

## Case presentation

**Figure 1:**
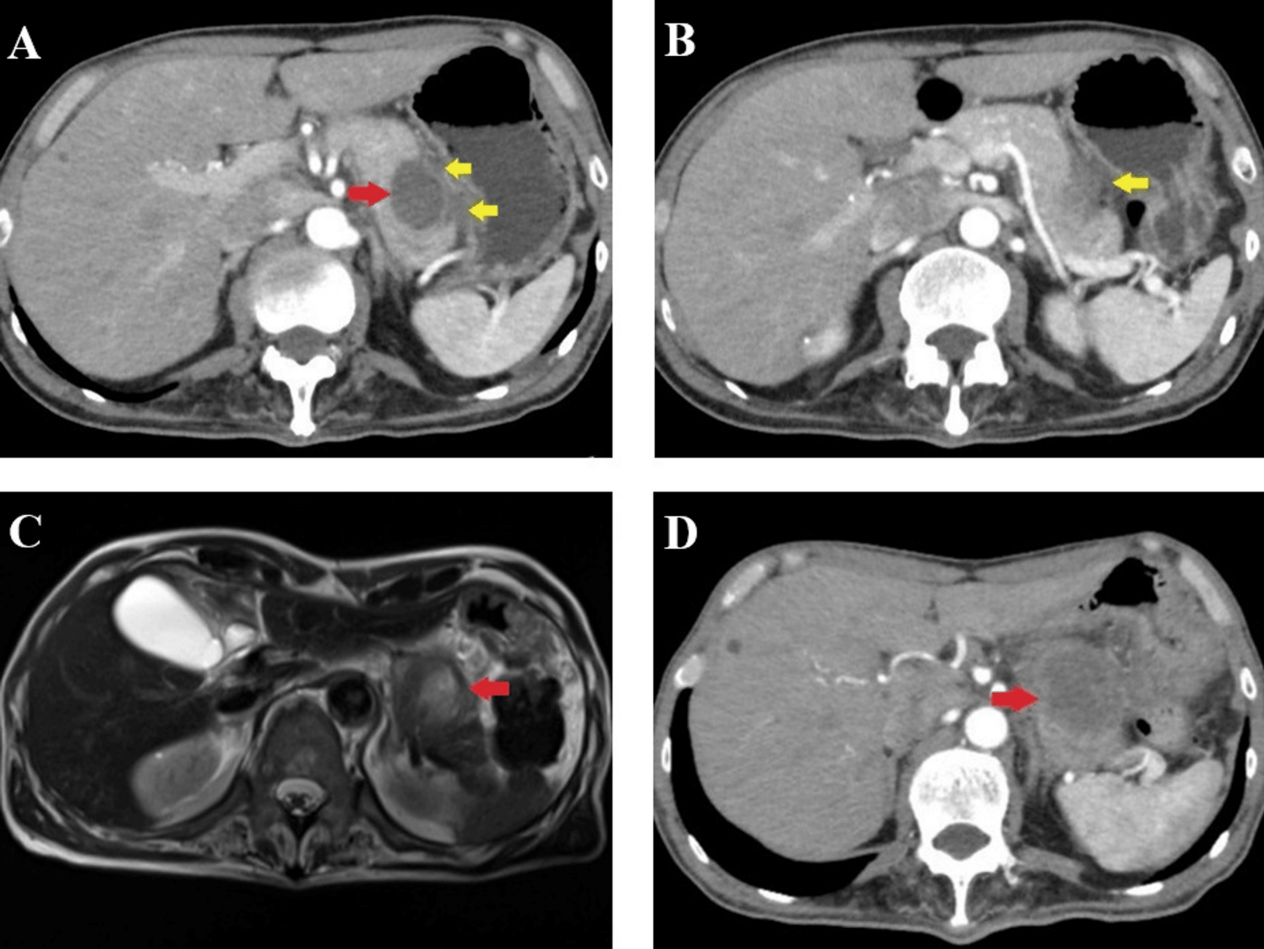
Imaging scans at the first examination. A: CT scan at initial presentation showing a well-defined low-density lesion localized to the pancreatic tail (red arrow) with a modest volume of peripancreatic fluid (yellow arrows). B: CT scan at initial presentation showing peripancreatic fluid and reduced signal intensity in the adjacent peripancreatic adipose tissue (yellow arrow). C: MRI imaging at initial presentation revealed a cyst showing heterogeneous high signal intensity on T1-weighted image (arrow). D: CT scan one month later showed enlargement of the lesion in the pancreatic tail (arrow).

A 63-year-old man visited our hospital complaining of upper abdominal pain and mild nausea that had persisted for one month. The patient had no history of pancreatitis, other malignancies, or previous abdominal surgery. Physical examination revealed tenderness in the upper abdomen without rebound tenderness or guarding. Laboratory tests revealed elevated inflammatory markers and a mild elevation of amylase levels. Tumor markers, including carcinoembryonic antigen (CEA) and carbohydrate antigen 19-9 (CA19-9), were within normal limits (Table 1). Abdominal ultrasonography revealed a cystic mass in the pancreatic tail. Subsequent contrast-enhanced computed tomography (CT) showed a well-defined low-density lesion measuring 30 mm in diameter, localized in the pancreatic tail (Figure 1A). A retention cyst secondary to pancreatitis was considered, based on the presence of peripancreatic fluid and diminished signal intensity in the surrounding adipose tissue (Figure 1B). Magnetic resonance imaging (MRI) revealed a cyst with heterogeneous high signal intensity on T1-weighted sequences and predominantly low signal intensity on T2-weighted sequences (Figure 1C). These imaging findings and an elevated serum C-reactive protein (CRP) level of 6.88 mg/dL (reference range: <0.3 mg/dL) suggested a pancreatic pseudocyst likely caused by pancreatitis. The normal amylase and lipase levels at presentation may reflect resolving pancreatitis, possibly due to prior antibiotic treatment prescribed at his previous medical facility.

**Table 1:** Laboratory test results (first outpatient visit). ↓ indicates values below the reference range. CRP: C-reactive protein; AST: aspartate aminotransferase; ALT: alanine aminotransferase; LD: lactate dehydrogenase; IFCC: International Federation of Clinical Chemistry; CEA: carcinoembryonic antigen; CA19-9: carbohydrate antigen 19-9; GOT: glutamic oxaloacetic transaminase; GPT: glutamic pyruvic transaminase.

Test	Value	Unit	Reference range
C-reactive protein (CRP)	6.88	mg/dL	<0.3
White blood cell count	7.7	×10³/μL	4.0–10.0
Red blood cell count	3.48	×10⁶/μL	4.5–5.9
Hemoglobin (Hb)	10.8	g/dL	13.5–17.5
Platelet count	308	×10³/μL	150–350
Albumin	3.5↓	g/dL	3.5–5
AST (GOT)	18	U/L	13–33
ALT (GPT)	17	U/L	8–42
LD (IFCC)	190	U/L	120–240
Total bilirubin	0.7	mg/dL	0.2–1.2
Alkaline phosphatase (IFCC)	71	U/L	38-113
Gamma-glutamyl transpeptidase	20	U/L	10–50
Creatinine (Cre)	1.05	mg/dL	0.7–1.3
CEA	0.8	U/mL	<5.0
CA19-9	8	ng/ml	<37
Amylase	108	U/L	25–125
Lipase	44	U/L	13–60
Elastase-1	419	ng/dL	<300

Although conservative management was initially employed, a follow-up CT one month later showed enlargement of the lesion (Figure 1D), and the CRP level was elevated to 10.28 mg/dL. Endoscopic ultrasonography (EUS) revealed a 40 mm hypoechoic lesion with internal linear hyperechoic components. No communication with the main pancreatic duct (MPD) was identified. The lesion was suspected to be a retention cyst secondary to occlusion of a branch of the pancreatic duct, and a small, latent pancreatic cancer was suspected as the underlying cause. EUS-guided fine-needle aspiration (FNA) was not performed because of concerns regarding the rupture of the cystic lesion.

**Figure 2:**
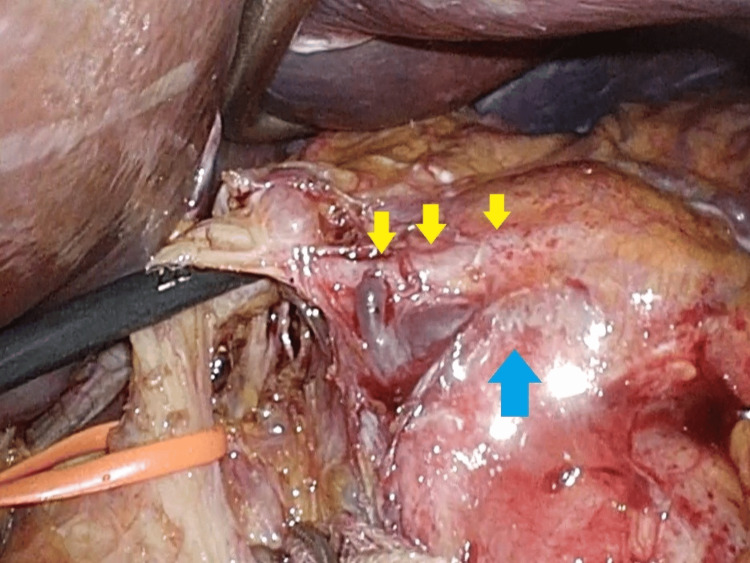
Intraoperative photograph. A mass extending from the pancreatic body to the tail (blue arrow), involving the left gastric artery (yellow arrows).

A laparoscopic distal pancreatectomy with splenectomy was therefore planned. Intraoperative findings showed no peritoneal disseminated lesions. A mass in the pancreatic body to the tail involved the left gastric artery. The lesion appeared more solid than cystic but was difficult to characterize macroscopically. Combined resection of the left gastric artery and surrounding soft tissue was performed to achieve a macroscopically clear margin (Figure 2). The pancreas was resected above the portal vein using a surgical stapler covered by a Neoveil (Endo GIA Reinforced Purple, Medtronic, Dublin, Ireland). A drain was placed near the pancreatic stump. Operative time was 284 minutes, and intraoperative blood loss was 230 g.

**Figure 3:**
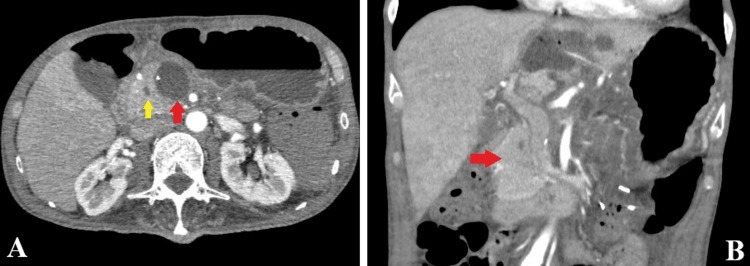
CT scan on postoperative day 20. A: Fluid collection around the pancreatic stump (red arrow) and dilation of the pancreatic duct up to 6 mm (yellow arrow). B: A small mass in the pancreatic head compressing the main pancreatic duct (red arrow).

The patient resumed oral intake on postoperative day (POD) three, although nausea and decreased appetite persisted. The drain was removed on POD five because of low amylase levels in the drainage fluid (156 U/L). The anorexia was suspected to be due to resection of the left gastric artery and disruption of the lesser curvature nerve plexus. CT on POD 14 showed fluid accumulation around the pancreatic stump and dilation of the pancreatic duct to 6 mm (Figure 3A). EUS was performed on POD 20, and encapsulated fluid surrounding the stump was aspirated via the gastric wall. The amylase level in the aspirate was high (39558 U/L), confirming the diagnosis of a pancreatic fistula. On POD 24, percutaneous drainage was performed, and a drain was inserted. The drainage fluid was clear, suggesting the presence of pure pancreatic fluid.

**Figure 4:**
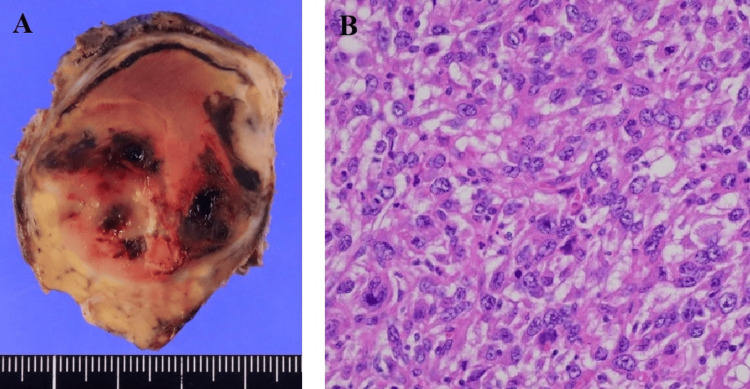
Macroscopic pathology and histopathology. A: Macroscopic pathology showing a mass lesion measuring 51 mm with cystic changes, extensive intertumoral hemorrhage, necrosis, and cavitary formation. B: Histopathology - Microscopically, the tumor consisted of a diffuse proliferation of pleomorphic and spindle-shaped cells.

**Figure 5:**
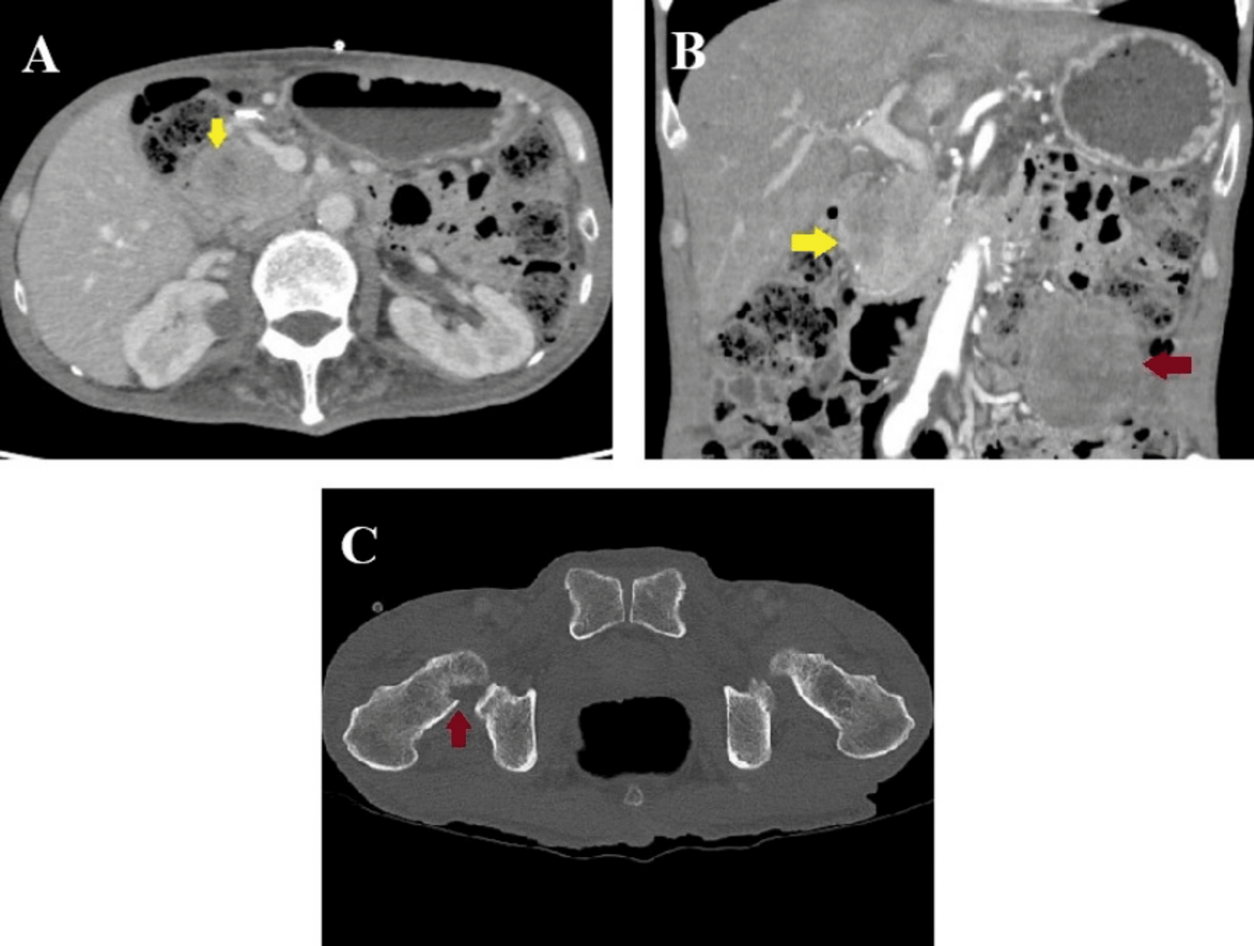
CT scan on postoperative day 53. A: A new mass replacing the remnant pancreas (yellow arrow). B: A new mass in the remnant pancreas (yellow arrow) and a peritoneal disseminated nodule (red arrow). C: Bone metastasis in the right femur (red arrow).

Pathological evaluation identified a mass lesion measuring 51 mm, with associated cystic changes, extensive intratumoral hemorrhage, necrosis, and cavitary formation (Figure 4A). Microscopically, the tumor exhibited a well-defined margin with variably sized enlarged nuclei and prominent spindle-shaped pleomorphic cells (Figure 4B). The final diagnosis was pleomorphic-type anaplastic carcinoma of the pancreas. Resection margins were negative for cancer, and lymph node metastases were negative. According to the Union for International Cancer Control (UICC) TNM (tumor, node, and metastasis) classification, the tumor was classified as pT2N0M0 stage IB. The drainage output persisted, and the patient was discharged on POD 39 with a percutaneous drain. At the first outpatient visit on POD 53, the drain output had decreased, and the percutaneous drain was removed. However, abdominal ultrasonography revealed a new mass in the lower abdomen. CT demonstrated a new mass that replaced the remnant pancreas, peritoneal dissemination, and bone metastasis to the right femur (Figure 5). Retrospective re-evaluation of the CT on POD 20 showed a small mass on the pancreatic head compressing the MPD (Figure 3B). The ductal stenosis of the pancreatic duct, which could be attributed to recurrence in the remnant pancreas, was considered the underlying cause of the delayed onset of pancreatic fistula, although fistulography via the drain had not been performed. The patient declined further oncological treatment, including chemotherapy, and opted for palliative care. He ultimately died of disease progression with multiple organ metastases and worsening general condition on POD 103.

## Discussion

Anaplastic carcinoma of the pancreas was first described as pleomorphic carcinoma by Sommers et al. in 1954, representing a rare subtype of pancreatic malignancy with sarcomatoid features [5]. In the World Health Organization (WHO) classification, undifferentiated carcinoma and anaplastic carcinoma are considered synonymous [6]. According to the Pancreatic Cancer Registry in Japan, the incidence of this tumor is exceedingly rare, accounting for only 0.14% (38 of 27,335 cases) of all pancreatic cancers [1]. The 7th edition of the General Rules for the Study of Pancreatic Cancer, published by the Japan Pancreatic Society (JPS), classifies anaplastic carcinoma into three subtypes: pleomorphic type, spindle cell type, and osteoclast-like giant cell type [7].

The present case was diagnosed as pleomorphic-type anaplastic carcinoma. This subtype is characterized by a predominance of spindle-shaped cells, accounting for about 80% of the cells without glandular formation and a growth pattern resembling sarcoma. Both mononuclear and multinucleated giant cells are also observed. Besides spindle-shaped cells, eosinophilic pleomorphic cells and regular round cells are often present [8]. In our case, diffuse proliferation of pleomorphic spindle-shaped polygonal cells infiltrating the surrounding tissue with enlarged nuclei and eosinophilic cytoplasm was observed.

Regarding tumor markers, several studies have reported that both CA19-9 and CEA can remain within normal limits [9-11]. Poorly differentiated tumors often lose glandular differentiation, which results in decreased production and secretion of epithelial tumor markers such as CA19-9 and CEA, contributing to their nonspecific preoperative levels. Tago et al. reported that preoperative CEA and CA19-9 levels were within normal ranges in 43.2% (19 of 44 cases) of patients with anaplastic carcinoma of the pancreas [12]. In our case, the absence of conventional pancreatic ductal adenocarcinoma components may explain the lack of tumor marker elevation, complicating the preoperative diagnosis.

Imaging findings of anaplastic carcinoma often include heterogeneous enhancement with dense peripheral contrast and poor central enhancement, owing to necrosis or hemorrhage [13]. No peripheral contrast enhancement was detected around the cystic lesion on preoperative imaging in our case, making it difficult to distinguish from a pseudocyst secondary to pancreatitis. Currently, the accuracy of preoperative diagnosis using imaging or tumor markers alone remains unsatisfactory. This highlights the need for additional diagnostic approaches. Although endoscopic ultrasound-guided fine-needle aspiration (EUS-FNA) cytology can be informative [14], its utility remains controversial due to concerns regarding diagnostic accuracy, risk of tumor dissemination, and bleeding, particularly in patients with suspected cystic lesions, such as in this case [15].

Anaplastic carcinomas are frequently detected as large masses, owing to their rapid progression during the clinical course. Extensive lymph node and multiple organ metastases are often present at the time of diagnosis [2]. In our case, the tumor was initially detected because of upper abdominal pain likely related to rapid tumor expansion, and early recurrence was observed in the remnant pancreas and distant organs. Anaplastic carcinoma is known for its infiltrative growth pattern, which often leads to obstruction of the main pancreatic duct and subsequent upstream dilatation. Although there was no postoperative pancreatic fistula (POPF) in the early postoperative period, it was assumed that the main pancreatic duct in the pancreatic head was obstructed by the growth of the recurrent tumor, leading to the rupture of the pancreatic stump on POD 14. Delayed onset of POPF is rare; its timing remains undefined, and its etiology has not been clearly established. According to previous reports, pancreatic duct obstruction, recurrent pancreatitis, and ischemia may contribute to anastomotic disruption, leading to delayed POPF [16]. In addition, the reason why the pancreatic fistula improved over the subsequent course is thought to be that the recurrent tumor in the remnant pancreas grew and eventually sealed the pancreatic stump.

Surgical resection is considered the only potentially curative treatment for anaplastic carcinoma [7,17,18]. However, even in resectable cases, the prognosis remains poor [8], due to the high incidence of local recurrence and distant metastasis following surgery. Local recurrence has been reported in 16.7% of anaplastic carcinoma cases after surgery [19]. Although surgery was performed due to the progressive enlargement of the mass, a repeat EUS examination might have facilitated a more accurate preoperative diagnosis. In retrospect, the possibility that distant metastases were already present at the time of surgery cannot be ruled out. Previous reports have indicated that positron emission tomography (PET) can show tracer accumulation along the cyst wall in such tumors [20]. Although PET-CT was not performed in this case, it might have contributed to a more definitive diagnosis. If the tumor had been identified as having an aggressive histology, PET-CT might have been considered to evaluate for distant metastases. Given the high metabolic activity of anaplastic carcinoma, PET-CT may also be superior to conventional imaging modalities in detecting occult metastases such as those in bone, lymph nodes, or other uncommon distant sites.

## Conclusions

We report a case of pleomorphic-type anaplastic carcinoma of the pancreas with early postoperative recurrence following distal pancreatectomy. Recurrence in the remnant pancreas can result in pancreatic stump disruption and the development of intractable pancreatic fistulas. Given the aggressive nature and diagnostic difficulty of this rare malignancy, a comprehensive diagnostic approach and a well-planned therapeutic strategy are essential.
